# Brain Responses to Dynamic Facial Expressions: A Normative Meta-Analysis

**DOI:** 10.3389/fnhum.2018.00227

**Published:** 2018-06-05

**Authors:** Oksana Zinchenko, Zachary A. Yaple, Marie Arsalidou

**Affiliations:** ^1^Centre for Cognition and Decision Making, Institute for Cognitive Neuroscience, National Research University Higher School of Economics, Moscow, Russia; ^2^Department of Psychology, National University of Singapore, Singapore, Singapore; ^3^Department of Psychology, National Research University Higher School of Economics, Moscow, Russia; ^4^Department of Psychology, York University, Toronto, ON, Canada

**Keywords:** dynamic faces, fMRI meta-analysis, activation likelihood estimate, social cognition, facial expressions

## Abstract

Identifying facial expressions is crucial for social interactions. Functional neuroimaging studies show that a set of brain areas, such as the fusiform gyrus and amygdala, become active when viewing emotional facial expressions. The majority of functional magnetic resonance imaging (fMRI) studies investigating face perception typically employ static images of faces. However, studies that use dynamic facial expressions (e.g., videos) are accumulating and suggest that a dynamic presentation may be more sensitive and ecologically valid for investigating faces. By using quantitative fMRI meta-analysis the present study examined concordance of brain regions associated with viewing dynamic facial expressions. We analyzed data from 216 participants that participated in 14 studies, which reported coordinates for 28 experiments. Our analysis revealed bilateral fusiform and middle temporal gyri, left amygdala, left declive of the cerebellum and the right inferior frontal gyrus. These regions are discussed in terms of their relation to models of face processing.

## Introduction

Effective face processing is essential for perceiving and recognizing intentions, emotion and mental states in others. Facial expressions have traditionally been investigated by utilizing static pictures of faces as opposed to dynamic moving faces (i.e., short video clips). Faces elicit activity in an established set of brain areas that includes the fusiform gyri associated with face perception, amygdala associated with processing affect and fronto-temporal regions associated with knowledge of a person (Fusar-Poli et al., 2009 for meta-analyses). Some suggest that dynamic faces compared to static faces are more ecologically valid (Bernstein and Yovel, 2015), and facilitate recognition of facial expressions (Ceccarini and Caudek, 2013). O'Toole et al. (2002) explain that when both static and dynamic identity information are available, people tend to rely primarily on static information for face recognition (i.e., supplemental information hypothesis), whereas dynamic information such as motion contributes to the quality of the structural information accessible from a human face (representation enhancement hypothesis). This dynamic information plays a key role in social interactions when evaluating the mood or intentions of others (Langton et al., 2000; O'Toole et al., 2002). The brain areas that respond to dynamic faces are not fully characterized with up-to-date meta-analysis methods and findings in the field. The purpose of this study is to examine concordance in brain regions associated with dynamic facial expressions using quantitative meta-analysis.

Functional magnetic resonance imaging (fMRI) studies investigating face perception typically reveal activation within the fusiform gyrus and occipital gyrus, areas part of the core regions of face processing, which mediate visual analysis of faces (O'Toole et al., 2002; Gobbini and Haxby, 2007). The extended system associated with extracting meaning from faces includes the inferior frontal cortex and amygdalae (Haxby et al., 2000). Notably, compared to static faces, much fewer fMRI studies use dynamic face stimuli, likely due to methodological and practical challenges in using dynamic faces. Specifically, short videos of faces need to be standardized in terms of presentation speed (i.e., how fast a neutral face transforms to an emotional expression), as this requires consistency across emotions. Similarly, morphed faces are modified to transform a static photo from a neutral to an emotional expression in a series of frames. Thus, adopting a protocol for using dynamic facial expressions (e.g., videos and morphs) requires more computational processing and in turn more time to prepare.

These additional efforts, however, have been found to be beneficial in populations that have an altered sensitivity to faces. For example, research shows that regions related to visual properties (i.e., the core system) and emotional/cognitive processing of faces (i.e., the extended system) are hypoactive in patients with autism spectrum disorders (Hadjikhani et al., 2007; Bookheimer et al., 2008; Nomi and Uddin, 2015 for review). Dynamic changes in facial expressions were used to show that individuals with and without autism spectrum disorders elicit equivalent activity in occipital regions, and differential activity in the fusiform gyrus, amygdala and superior temporal sulcus, suggesting a dysfunction in the relational and affective processing of faces (Pelphrey et al., 2007). Thus, in practice, usage of dynamic stimuli would be advantageous when studying populations with difficulties in processing faces and emotions.

A recent review of the face perception literature adopted the model of core and extended systems to explain processing of dynamic faces in typical adults (Bernstein and Yovel, 2015). This review provides support for a dorsal stream that encompasses the superior temporal sulcus, and encodes low-frequency information such as face motion, head rotation and processing of moving facial parts (O'Toole et al., 2002; Peyrin et al., 2004, 2005, 2010; Saxe, 2006), and a ventral stream that comprises bilateral inferior occipital cortex and fusiform gyrus, and processes high-frequency information such as facial expressions and face parts (e.g., Eger et al., 2004; Iidaka et al., 2004; Corradi-Dell'Acqua et al., 2014). Since the dorsal stream processes more information about movement of faces, dynamic facial expressions should involve more activation of the superior temporal lobe.

An early meta-analysis analyzed coordinates from 11 experiments on dynamic facial expressions and identified concordance in temporal, parietal, and frontal cortices (Arsalidou et al., 2011). Since then, there has been an increase in the number of fMRI studies that examine brain responses to dynamic faces. Critically, there have been methodological advances to the activation likelihood estimation (ALE) method (Turkeltaub et al., 2012) and documented implementation errors in the old ALE software that have since been corrected (Eickhoff et al., 2017); ALE software developers recommend re-analyses and evaluation of current and past meta-analyses. Thus, the purpose of the current paper was to examine brain areas associated with processing of dynamic facial expressions in healthy adults and establish their implication above and beyond to brain areas responding to static faces and other control tasks.

## Methods

### Literature search and article selection

**Figure 1:**
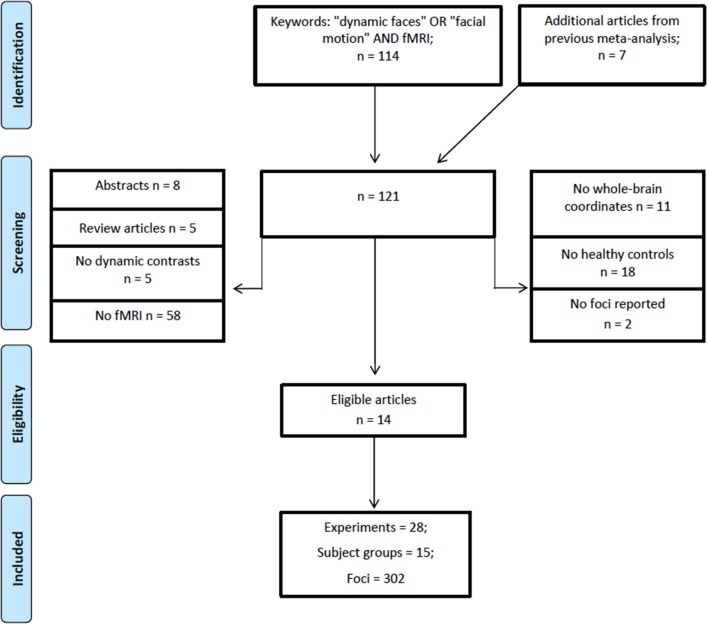
PRISMA flowchart for eligibility of articles (Template by Moher et al., 2009).

A literature search was performed using Web of Science (http://apps.webofknowledge.com/) on October, 6th, 2017, keywords (“dynamic faces” OR “facial motion” AND “fMRI”), years 1995–2017, yielding a total of 114 articles. Figure 1 shows the steps taken to identify eligible articles. Specifically, we excluded articles that: (1) reported no fMRI data; (2) studies that did not report whole brain analysis; (3) reported no data on healthy adults; (4) did not report fMRI coordinates and, (5) articles with irrelevant tasks. Articles surviving these criteria underwent a full text review by two researchers independently (O.Z. and Z.Y.). The remaining articles included healthy adults; reported stereotaxic coordinates in Talairach or Montreal Neurological Institute (MNI) space from random effects whole-brain analysis, which reported a contrast (i.e., experiment) comparing dynamic with static faces. Articles from a previous meta-analysis and an eligible study within it (Arsalidou et al., 2011) resulted in 7 additional articles. All relevant experiments from each article were included in the analysis because the most recent algorithm uses a correction to avoid summation of within-group effects and provides increased power (Turkeltaub et al., 2012). Table 1 shows participant demographics and details from a total 28 experiments from 14 articles, sorted by 15 separate subject groups, which were included in the meta-analysis. The number of experiments we included in the analysis adheres to current recommendations (*n* = 17–20) for achieving sufficient statistical power (Eickhoff et al., 2017).

**Table 1:** Descriptive information of studies and contrasts used in the meta-analyses.

**Article**	***n***	**Male**	**Handedness**	**Age range**	**Task**	**Contrast**	**Foci**	***p*-value^c^**
Arsalidou et al., 2011	15	2	N/A	26.3 ± 4.5	Dynamic and static happy and neutral^a^	Happy: dynamic > static	2	*P* < 0.01 using cluster level threshold *p* = 0.05
						Dynamic > static	5	*P* < 0.01, using cluster level threshold *p* = 0.05
Grosbras and Paus, 2006	20	10	R	19–46	Angry and neutral movements of faces^b^	Neutral: dynamic > control	28	*P* < 0.05 using Gaussian random-field theory to correct for multiple comparisons
						Angry: dynamic > control	27	*P* < 0.05 using Gaussian random-field theory to correct for multiple comparisons
Hurlemann et al., 2008	14	7	R	25.04 ± 2.4	Dynamic happy and angry facial animations^b^	Dynamic:emotional > neutral	10	*P* < 0.001, uncorrected
						Dynamic: angry > neutral	3	
						Dynamic: happy > neutral	17	
LaBar et al., 2003	10	5	R	21–30	Dynamic and static; angry and fearful^b^	Anger morph > static	6	*P* < 0.001, uncorrected
						Fear morph > static	16	
						Identity morph > static neutral	16	
						Emotion morph > static emotion	17	
Lee et al., 2010	17	7	R	24.94 ± 4.16	Dynamic and static turning head^a^	Turning heads > static heads	9	*P* < 0.05, cluster corrected,
Pelphrey et al., 2007	8	6	All R	24.1 ± 5.6	Dynamic and static; angry and fearful^a^	Dynamic emotions > static emotions (normal group)	6	*P* < 0.05, uncorrected
Pentón et al., 2010	13	8	N/A	19–55	Static and dynamic, neutral and fearful faces^b^	Dynamic > static	21	*P* < 0.05, FDR corrected
Robins et al., 2009	10	3	N/A	22.3 ± 4.6	Dynamic angry, happy, fearful, and neutral^b^	Dynamic emotion > neutral	5	*P* < 0.001
Sarkheil et al., 2013	20	9	R	20–42	Angry and happy morph face stimuli^b^	Intensity effect (more > less)	8	*P* < 0.05, cluster-size thresholding
Sato et al., 2015	15	9	R	26.9 ± 3.9	Fearful, happy, and neutral dynamic and static faces and mosaics^a^	Dynamic facial > dynamic mosaics; time 150–200	13	*P* < 0.05 corrected for multiple comparisons with a height threshold of *P* < 0.01 (uncorrected)
						Dynamic facial > dynamic mosaics; time 200–250	3	
						Dynamic facial > dynamic mosaics; time 250–300	4	
						Dynamic facial > dynamic mosaics; time 300–350	6	
						Dynamic facial > dynamic mosaics; time 350–400	5	
Sato et al., 2004	11	^*^	R	26.5	Dynamic fearful and neutral faces^a^	Fear: dynamic > static	18	*P* < 0.05
	11	^*^				Happy: dynamic > static	12	
Schultz and Pilz, 2009	10	6	N/A	N/A	Dynamic and static; angry and surprised^b^	Dynamic faces > static faces	6	*P* < 0.05, FDR-corrected and cluster-wise corrected
Schultz et al., 2013	26	14	R	22–39	Video recordings of moving faces, static faces and scrambled order of dynamic faces^b^	Movies with ordered frames > movies with scrambled frames	3	*P* < 0.001, uncorrected
						Original 25 Hz movies > static faces	4	
Trautmann et al., 2009	16	0	R	21.6 ± 2.3	Dynamic and static; happy and disgust^a^	Dynamic faces (happy > neutral) > static faces (happy > neutral)	14	*P* < 0.001, uncorrected
						Dynamic faces (disgust > neutral) > static faces (disgust > neutral)	18	

*n = sample size*;

* = *22 participants (10 males) participated in two studies, gender assignment was not specified; N/A, not available; R, all right handed*;

a *studies that instruct participants to passively view faces*;

b *studies that instruct participants to make judgments about faces*,

c *thresholding settings reported in paper*.

### Meta-analysis

The meta-analysis was performed using GingerALE software (2.3.6), which relies on ALE, a coordinate-based meta-analytic method (Eickhoff et al., 2009, 2017) available at http://www.brainmap.org/ale/. Foci from different articles were used to create a probabilistic map that compares the likelihood of activation compared to random spatial distribution. MNI coordinates were converted to Talairach space using the Lancaster et al. (2007) transformation. Significance was assessed using a cluster-level threshold for multiple comparisons at *p* = 0.05 with a cluster-forming threshold set to *p* = 0.001 (Eickhoff et al., 2012, 2017). GingerALE software does not provide an option for estimating replicability of the data, however, based on simulations of ALE analyses that have been performed to test sensitivity, number of incidental clusters and statistical power (Eickhoff et al., 2016), a recommended minimum number of experiments (*N* = 17–20) has been proposed (Eickhoff et al., 2017). Moreover, a cluster-level threshold sets the cluster minimum volume such that only, for example, 5% of the simulated data clusters exceed this size, minimizing the possibility that an ALE peak could be driven by only one study.

The majority of studies used tasks where participants were instructed to passively observe facial stimuli (Sato et al., 2004; Trautmann et al., 2009; Pentón et al., 2010; Arsalidou et al., 2011) or to perform a simple target detection task (Pelphrey et al., 2007; Robins et al., 2009; Lee et al., 2010; Sato et al., 2015). Two studies asked to rank the presented emotional expressions (Grosbras and Paus, 2006; Sarkheil et al., 2013); three studies instructed the participants to make a decision about the gender of face stimuli (Hurlemann et al., 2008; Pentón et al., 2010; Ceccarini and Caudek, 2013); one study asked to rank the meaningfulness of moving faces and judge the fluidity of facial motions (Schultz et al., 2013); in another study participants were told to identify the category of face stimuli (LaBar et al., 2003); and in another study participants performed a one-back matching task (Schultz and Pilz, 2009). Five articles reported experiments related to dynamic > static in various emotions: anger (LaBar et al., 2003; Grosbras and Paus, 2006), fear (Sato et al., 2004), and happiness (Sato et al., 2004; Trautmann et al., 2009; Arsalidou et al., 2011). Six articles presented participants with dynamic > static faces after subtracting neutral from emotional faces in one (Hurlemann et al., 2008), several (Pelphrey et al., 2007; Robins et al., 2009; Schultz and Pilz, 2009), or no emotional component (Lee et al., 2010; Pentón et al., 2010). One article reported experiments regarding the morph intensity effect in dynamic faces (Sarkheil et al., 2013), and two articles contrasted dynamic faces to mosaic stimuli (Sato et al., 2015; we note that this study reported fMRI coordinates using magnetic encephalography-fMRI data reconstruction) or scrambled faces (Schultz et al., 2013).

## Results

Analyses included data from 216 right-handed participants (27.24 ± 9.02 years; 39.81% men, Table 1 for details).

### ALE map

**Figure 2:**
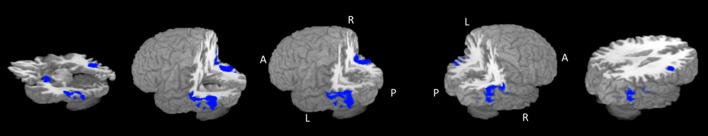
Rendered ALE map showing significant concordance across studies for dynamic facial expressions. A, anterior; P, Posterior; L, left; R, right. All coordinates are listed in Table 2.

The largest cluster with the highest ALE value was found in the right hemisphere and extended from the inferior temporal and occipital, to fusiform and superior temporal gyri (Figure 2, Table 2). The second cluster was found in left hemisphere and extended from the middle occipital and temporal gyri to the fusiform gyrus and cerebellum. Other areas included the left amygdala, and right inferior frontal gyrus.

**Table 2:** Concordant brain regions associated with dynamic facial expressions.

		**Talairach**			
**Volume mm^3^**	**ALE Value**	**x**	**y**	**z**	**Brain Area**
9,256	0.0395	42	−64	2	Right Inferior Temporal Gyrus
	0.0366	44	−60	−6	Right Inferior Temporal Gyrus BA 19
	0.0282	40	−56	−16	Right Fusiform Gyrus BA 37
	0.0256	54	−44	4	Right Middle Temporal Gyrus BA 22
	0.0138	56	−42	18	Right Superior Temporal Gyrus BA 13
	0.0130	40	−78	−2	Right Inferior Occipital Gyrus BA 19
8,480	0.0389	−42	−70	2	Left Middle Occipital Gyrus BA 37
	0.0216	−50	−44	4	Left Middle Temporal Gyrus BA 22
	0.0199	−44	−46	−16	Left Fusiform Gyrus BA 37
	0.0197	−40	−64	−8	Left Fusiform Gyrus BA 19
	0.0190	−38	−56	−18	Left Cerebellum, Declive
	0.0185	−50	−58	4	Left Middle Temporal Gyrus BA 37
	0.0135	−46	−72	−14	Left Fusiform Gyrus BA 19
1,160	0.0188	−16	−6	−12	Left Amygdala
840	0.0234	48	4	24	Right Inferior Frontal Gyrus BA 9

## Discussion

We examined concordance across studies in brain areas responding more to dynamic facial expressions. We report concordance in: (a) areas associated with the core visual system of processing faces such as fusiform gyrus and posterior parts of the superior temporal gyrus, (b) areas associated with the extended system for processing faces such as the left amygdala, inferior frontal gyrus, and anterior parts of the superior temporal gyrus and (c) a cluster within the cerebellar declive, a region previously not highlighted in models of facial cognition. We build on previous models of face processing and discuss possible roles of these areas during the processing of dynamic faces.

In comparison with the previous meta-analysis on dynamic faces (Arsalidou et al., 2011); the current analysis yields similar brain regions, however the output resulted in less clusters that were larger in size and carried higher ALE values. When comparing the top clusters, the amygdala and cerebellar declive are found in the left hemisphere for both the current and previous analyses. Clusters in right precuneus (BA 7) and cuneus, and left hypothalamus, previously found to be concordant (Arsalidou et al., 2011), were not observed in the current meta-analysis; these areas had both lower ALE scores and smaller cluster volumes. We note three methodological choices that may account for differences in the current and previous meta-analyses; (a) the number of experiments included in the current meta-analyses is larger, which provide increased power, (b) the GingerALE algorithm, which allows for controlling for within-group effects and provides increased power (Turkeltaub et al., 2012) and (c) the thresholding approach follows cluster-level threshold for controlling for multiple comparisons, which is more suitable for ALE meta-analyses (Eickhoff et al., 2016, 2017). Critically, the current meta-analysis shows that the overall size of clusters in occipito-temporal regions is similar in the right and left hemisphere, suggesting bilateral engagement.

Specifically, bilateral occipito-temporal gyri comprise of the fusiform and superior temporal gyri, areas are most associated with face processing; the fusiform gyri are implicated in configuring relations among visual features and relying on high-spatial-frequency to form face percepts as a whole (e.g., Vuilleumier et al., 2003; Iidaka et al., 2004; Sabatinelli et al., 2011), or in part (e.g., Rossion et al., 2003; Nichols et al., 2010; Yaple et al., 2016). This is consistent with models that classify the fusiform gyrus as part of the core visual processing system for faces (Gobbini and Haxby, 2007), and as part of the ventral stream of face processing (e.g., Bernstein and Yovel, 2015).

Moreover, we observe concordance in posterior and more dorsal parts of the superior temporal gyri. The superior temporal gyri are known for their involvement in the analysis of low-spatial frequency information (i.e., global facial information) such as gaze direction and motion associated with interpreting social signals (Allison et al., 2000; Taylor et al., 2009; Wegrzyn et al., 2015). According to the face perception model by Haxby and colleagues posterior parts of the superior temporal sulcus are part of the core visual face processing system responsible for basic visual analyses of faces, whereas adjacent more anterior parts of the superior temporal gyri are part of the extended system that is responsible for further processing of personal information (Haxby et al., 2000; Gobbini and Haxby, 2007). Our data are also consistent with the more recent interpretation of a dorsal face processing pathway proposed by Bernstein and Yovel (2015). Importantly, consistent with the representation enhancement hypothesis (O'Toole et al., 2002) we propose that dynamic faces may show increased implication in superior temporal cortices because they provide richer input for the brain to interpret.

As part of the left occipito-temporal cluster we observed concordance in the cerebellar declive, an area not highlighted as part of face processing models. Traditionally, the cerebellum was known for its involvement in motor functioning. However, its role in cognitive and affective processing has been discussed (e.g., Brooks, 1984; Paulin, 1993; Doya, 2000; Stoodley and Schmahmann, 2010) and a generic role in timing mechanisms has been proposed (e.g., Ivry and Spencer, 2004). Past meta-analyses identify concordance in the cerebellum for static facial expressions (Fusar-Poli et al., 2009), however its role in social cognition remains unclear. In relation to social processes some have shown that the cerebellum is associated with mirroring and mentalizing motor actions (Van Overwalle et al., 2014, 2015). We suggest that the cerebellum may play a role in tracking the sequences for conveying the signal and updating the information about perceptual features in a face to predict possible changes, similar to its involvement in the motor system.

Concordance in the left amygdala and right inferior frontal gyrus is respectively associated with emotional and cognitive processing of faces. The amygdala responds to all sorts of emotional stimuli such as fear processing and fear conditioning (LeDoux, 2003), reward and punishment (Gupta et al., 2011). Growing evidence suggests that amygdala activation is not specific to fearful expressions or any particular emotion (van der Gaag et al., 2007), but rather it processes salient information of faces (Fitzgerald et al., 2006). It has been suggested that the amygdala contribute to social-emotional recognition (Adolphs et al., 2002; Adolphs and Spezio, 2006) and processing of salient face stimuli during unpredictable situations (Adolphs, 2010). Some have emphasized the evolutionary significance of the amygdalae, suggesting it plays a role in detecting relevant stimuli (Sander et al., 2003) and signaling potentially significant consequential events (Fitzgerald et al., 2006). Thus, based on past findings, perhaps the processing of dynamic faces requires increased amygdala activation due to an increased vigilance in observing the dynamically changing salient features of faces.

The inferior frontal gyrus, a part of the ventrolateral prefrontal cortex, is associated with all sorts of cognitive functions including response inhibition (Aron et al., 2003; Hampshire et al., 2009, 2010), working memory (Yaple and Arsalidou, in press), negative priming (Yaple and Arsalidou, 2017) and mental attention (Arsalidou et al., 2013). A hierarchical model of the prefrontal cortex suggests that the inferior frontal gyri would be responsible for simple, non-abstract judgments (Christoff et al., 2009). The majority of studies asked participants to make simple judgments about gender, emotion, or motion of faces congruent with this hypothesis. Regarding right lateralization, relevant to social interactions, the right inferior frontal gyrus is active when processing social information such as cooperative interaction (Liu et al., 2015) and interpersonal interactions (Liu et al., 2016). It has been shown that bilateral inferior frontal gyrus as a part of the dorsomedial network (Bzdok et al., 2013), which is involved in contemplation of others' mental states (Mar, 2011 for meta-analysis). Alternatively, based on a trade-off between task difficulty and the mental-attentional capacity of the individual, the right hemisphere is hypothesized to be favored in simple, automatized processes (Pascual-Leone, 1989; Arsalidou et al., 2018 for details). Overall, right inferior frontal gyrus's activation during face perception may be associated with cognitive processing of social information processing or maintaining with simple task requirements.

## Limitations

Data presented here represent concordance across fMRI studies that investigated dynamic vs. static facial expressions and across different emotional states. ALE methodological limitations have been discussed elsewhere (Zinchenko and Arsalidou, 2018; Yaple and Arsalidou, in press) and include lack of control of statistical methodologies adopted by original articles and consideration only of peak coordinates. A shortcoming of the current study is data we report here are in majority based on female participants as original articles favored recruiting female participants who may show a greater response to faces.

## Conclusion

A coordinate-based meta-analysis was performed to assess the concordance of brain activations derived from experiments that identified more activity in dynamic compared to static faces and other control tasks. We observed concordance across studies in brain areas well established in the face processing literature, as well as the cerebellum, which is not discussed in models associated with face processing. The observed results suggest that dynamic faces require increased resources in the brain to process complex, dynamically changing features of faces. The current data provide a stereotaxic set of brain regions that underlie dynamic facial expression in typical adults. Practically, these normative data can serve as a benchmark for future studies with atypical populations, such as individuals with autism spectrum disorder. Theoretically, these findings provide further support for an extended set of areas that support processing of dynamic facial expression. Overall, our present findings can inform current models and help guide future studies on dynamic facial expressions.

## Author contributions

OZ helped collect and analyze data and prepared the first draft of the manuscript. ZY helped collect and analyze data and contributed to manuscript preparation. MA conceptualized research and contributed to manuscript preparation.

### Conflict of interest statement

The authors declare that the research was conducted in the absence of any commercial or financial relationships that could be construed as a potential conflict of interest.
